# Rewiring an olfactory circuit by altering the combinatorial code of cell-surface proteins

**DOI:** 10.21203/rs.3.rs-6099298/v1

**Published:** 2025-03-14

**Authors:** Cheng Lyu, Zhuoran Li, Chuanyun Xu, Jordan Kalai, Liqun Luo

**Affiliations:** 1 Department of Biology and Howard Hughes Medical Institute, Stanford University, Stanford, CA 94305, USA; 2 Biology Graduate Program, Stanford University, Stanford, CA 94305, USA

## Abstract

Proper brain function requires the precise assembly of neural circuits during development. Despite the identification of many cell-surface proteins (CSPs) that help guide axons to their targets^1,2^, it remains largely unknown how multiple CSPs work together to assemble a functional circuit. Here, we used synaptic partner matching in the *Drosophila* olfactory circuit^3,4^ to address this question. By systematically altering the combination of differentially expressed CSPs in a single olfactory receptor neuron (ORN) type, which senses a male pheromone that inhibits male-male courtship, we switched its connection from its endogenous postsynaptic projection neuron (PN) type nearly completely to a new PN type that promotes courtship. To achieve this switch, we deduced a combinatorial code including CSPs that mediate both attractive and repulsive interactions between synaptic partners^5,6^. The anatomical switch changed the odor response of the new PN partner and markedly increased male-male courtship. We generalized three manipulation strategies from this rewiring to successfully rewire a second ORN type to multiple distinct PN types. This work demonstrates that manipulating a small set of CSPs is sufficient to respecify synaptic connections, paving ways to explore how neural systems evolve through changes of circuit connectivity.

## Main text

The precise wiring of neural circuits is the foundation of brain function. In his chemoaffinity hypothesis, Sperry speculated that “brain cells and fibers must carry some kind of individual identification tags, by which they are distinguished one from another almost, in many regions, to the level of a single neuron”^7^. Many CSPs have since been identified that guide axons to specific brain regions^1,2^. CSPs that instruct synaptic partner selection within a specific brain region have also begun to be identified^8^. However, disrupting individual CSPs, even with complete loss-of-function mutations, usually leads to partial phenotypes at specific wiring steps, particularly in synaptic partner selection^5,6,9^, suggesting considerable redundancy. While redundancy could, in principle, increase the robustness of circuit wiring^3^, it poses technical challenges to using a reductionist approach to achieve a complete understanding of how different CSPs work together to assemble a functional circuit, a central goal of developmental neurobiology.

An alternative approach to understanding circuit assembly is to re-engineer the combinatorial expression of CSPs in a single neuron type, with the aim of completely rewiring these neurons away from their endogenous synaptic partner and to a new partner. One of the main challenges of rewiring a neural circuit is that the number of CSPs needed is suspected to be large in general^8^. Here, we report taking such an approach in the *Drosophila* olfactory circuit.

In adult *Drosophila*, about 50 types of ORNs form one-to-one synaptic connection with 50 types of PNs at 50 discrete glomeruli, providing an excellent system for studying mechanisms underlying synaptic partner matching. Several recent studies have motivated our attempts to rewire the fly olfactory circuits. First, despite the 3-dimensional organization of 50 glomeruli in adults, during development, each ORN axon only needs to search for synaptic partners along a 1D trajectory on the surface of the antennal lobe^10^. This greatly reduces the number of synaptic partners from which individual ORN axons need to distinguish. Second, examining ORN axon development at single-neuron resolution revealed that each ORN axon extends multiple transient branches along its trajectory in early stages of development and branches that contact partner dendrites are selectively stabilized^4^. Third, in a companion manuscript^5^, we report the identification of three CSP pairs that signal repulsion during development to prevent synaptic connections between non-cognate ORN and PN pairs. These repulsive CSPs, along with several attractive CSPs previously characterized^6^ or reported here, are key components in the combinatorial codes for synaptic partner matching we are about to describe.

## Genetic tools to visualize rewiring

**Extended Data Fig. 1 |:**
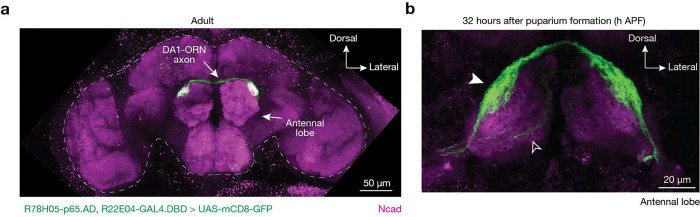
DA1-ORN split-GAL4 characterization. **a**, In adults, the split-GAL4, R78H05-AD+R22E04-DBD, labels DA1-ORNs in the whole brain as revealed by GFP staining (green). The brain border is dashed outlined. **b**, At around 32h APF, the same split-GAL4 driver most strongly labels DA1-ORNs (solid arrowhead) and weakly and sparsely labels a few other ORN types whose axons take the ventromedial trajectory (open arrowhead). Magenta: N-cadherin (Ncad) staining for neuropils. Maximum z-projection is shown.

**Fig. 1 |:**
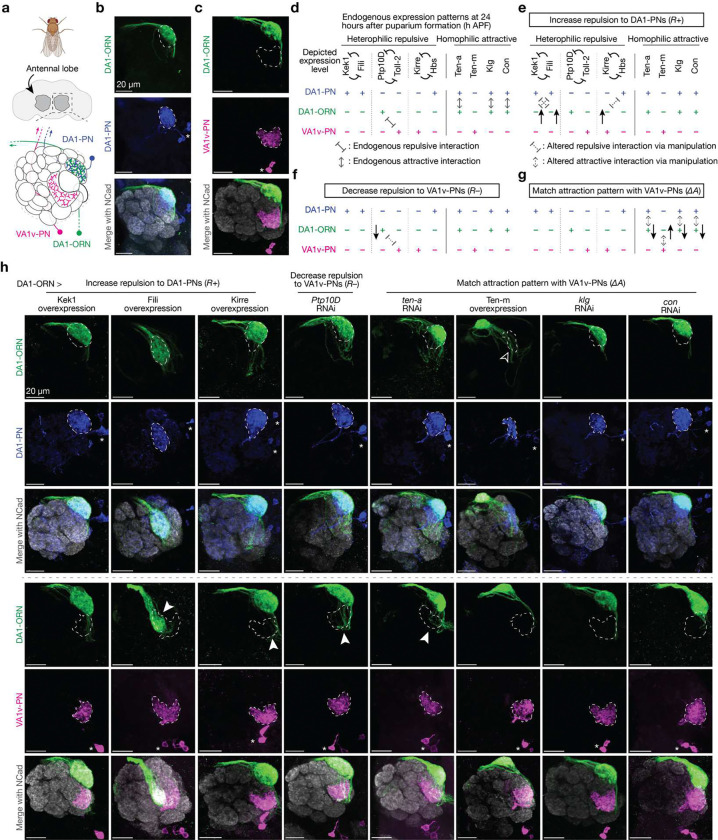
Manipulating single CSPs in DA1-ORNs produces minor DA1-ORNs→VA1v-PNs rewiring. **a**, Adult *Drosophila* brain and antennal lobe schematics. DA1-ORN axons (green) match with DA1-PN dendrites (blue), but not with VA1v-PN dendrites (magenta). Same color code in all other panels. **b**, Maximum z-projection of adult antennal lobes around DA1-ORN axons (green, labeled by a membrane-targeted GFP driven by a split-GAL4) and DA1-PN dendrites (blue, labeled by a membrane-targeted RFP driven by a QF2 driver). DA1 glomerular border (dashed outlined) determined by N-cadherin (Ncad) staining. * designates PN cell bodies, and scale bar = 20 μm (all panels). **c**, Same as (**b**), but with VA1v-PN dendrites (magenta) labeled instead of DA1-PN dendrites. VA1v glomerular border dashed outlined. **d**, Summary of expression levels of the ten CSPs considered in the rewiring experiments. ‘+’ indicates high expression level and ‘−’ indicates low expression level. The expression levels are largely inferred from the previously collected single-cell RNA sequencing dataset, and are confirmed or corrected with the protein data when available (Extended Data Fig. 3). 24h APF is a developmental stage just prior to the onset of synaptic partner selection via stabilization of transient ORN axon branches^4^. The three pairs of CSPs on the left mediate heterophilic repulsive interaction. The four CSPs on the right signal homophilic attractive interaction. Here, we omitted Sns and only considered Kirre and Hbs in our analysis since Sns is lowly expressed in all neurons involved in our rewiring experiments (Extended Data Fig. 3). **e**, Proposed genetic manipulations to increase the repulsion between DA1-ORN axons and DA1-PN dendrites during development. Black arrows in panels e–g indicate proposed genetic manipulations in DA1-ORNs, with upward and downward arrows for overexpression and RNAi knockdown, respectively. **f**, Same as (**e**), but on proposed genetic manipulations to decrease the repulsion between DA1-ORN axons and VA1v-PN dendrites. **g**, Same as (**e**), but on proposed genetic manipulations to match the attraction between DA1-ORN axons and VA1v-PN dendrites. **h**, Rewiring effects when CSPs are manipulated separately. Genetic manipulations are labeled on the top. Maximum z-projections of adult antennal lobes around DA1-ORN axons are shown. Top three rows: DA1-PNs are co-labeled with borders outlined. The open arrowhead indicates the decrease of overlap between DA1-ORN axons and DA1-PN dendrites. Bottom three rows (different brains from the top three rows): VA1v-PNs are co-labeled with borders outlined. Arrowheads indicate the mismatch of DA1-ORN axons with VA1v-PN dendrites. Overlapping ratios are quantified in Fig. 2a.

**Fig. 2 |:**
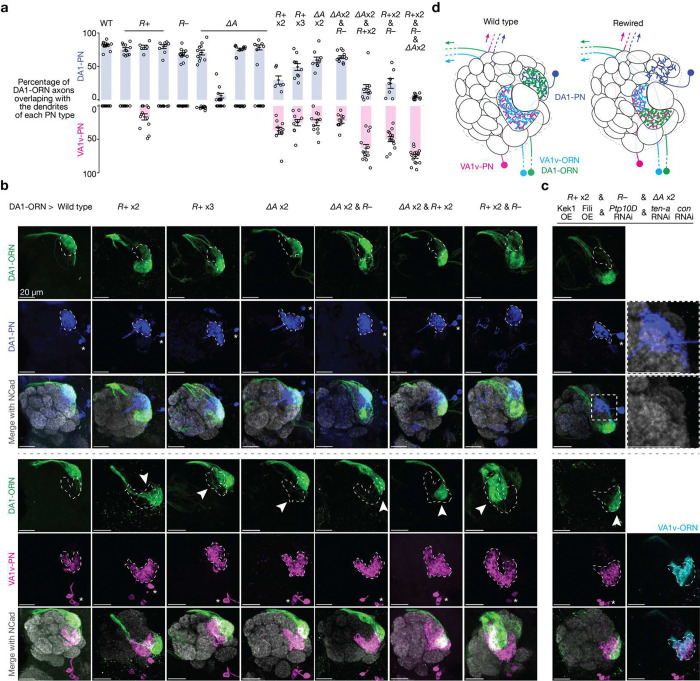
Simultaneous altering the expression of five CSPs in DA1-ORNs causes nearly complete DA1-ORNs→VA1v-PNs rewiring. **a**, Percentage of DA1-ORN axons overlapping with the dendrites of DA1-PNs (top) and VA1v-PNs (bottom). Circles indicate individual antennal lobes; bars indicate the population mean ± s.e.m. ‘*R*+’: Kek1 overexpression (OE), Fili OE, and Kirre OE (left to right). ‘*R*−’: *Ptp10D* RNAi. ‘Δ*A*’: *ten-a* RNAi, Ten-m OE, *klg* RNAi, and *con* RNAi (left to right). ‘*R*+ x2’: Kek1 OE + Fili OE. ‘*R*+ x3’: Kek1 OE + Fili OE + Kirre OE. ‘Δ*A* x2’: *ten-a* RNAi + *con* RNAi. Same labels used in (**b**). **b**, Rewiring effects when CSPs are manipulated combinatorially. Genetic manipulations are labeled on the top. Maximum z-projections of adult antennal lobes around DA1-ORN axons (green) are shown. Top three rows: DA1-PNs (blue) are co-labeled with borders dashed outlined. Bottom three rows: VA1v-PNs (magenta) are co-labeled with borders dashed outlined. Arrowheads indicate the mismatch of DA1-ORN axons with VA1v-PN dendrites; Scale bar = 20 μm; * designates PN cell bodies; Overlapping ratios are quantified in (**a**). The leftmost column is repeated from Fig. 1b, c for easy comparison within this panel. **c**, Same as (**b**), but with all three manipulation strategies combined. Two images on the top of the right column are zoom-ins from the dashed squares to the left. Two images on the bottom of the right column are from the same brain as in the left column, but with VA1v-ORNs co-labeled (cyan, *Or47b* promotor driven rat CD2). **d**, Schematics of DA1-ORNs and -PNs as well as VA1v-ORNs and -PNs in the wild-type (left) and DA1-ORN rewired (right) antennal lobe. In the rewired lobe: axons of DA1-ORNs and VA1v-ORNs split VA1v-PN dendrites; DA1-PN dendrites spread into multiple adjacent glomeruli.

We first sought to rewire ORNs that normally target their axons to the DA1 glomerulus (DA1-ORNs) to instead synapse with VA1v-PNs, whose dendrites tile the VA1v glomerulus (Fig. 1a), by combinatorially manipulating the expression levels of different CSPs in DA1-ORNs. We chose these two glomeruli because the axons of both DA1-ORNs and VA1v-ORNs take similar trajectory during development^11^ and because they process signals that have the opposite effects on male courtship activity^12,13^ (see details below). To simultaneously manipulate the expression levels of multiple CSPs specifically in DA1-ORNs during the wiring process, we generated a genetic driver that specifically labels DA1-ORNs across developmental stages using split-GAL4^14^ (hereafter DA1-ORN driver, Extended Data Fig. 1). To examine the matching of DA1-ORN axons with the dendrites of either DA1-PNs or VA1v-PNs in adults, we co-labeled DA1-ORNs (using the split-GAL4 above) with either DA1-PNs or VA1v-PNs in the same adult brain using the orthogonal QF/QUAS^15^ and LexA/LexAop^16^ systems, respectively (Fig. 1b, c). In wild-type flies, DA1-ORN axons overlapped with DA1-PN dendrites but not with VA1v-PN dendrites (Figs. 1b, c, 2a).

## Three manipulation strategies for rewiring

**Extended Data Fig. 2 |:**
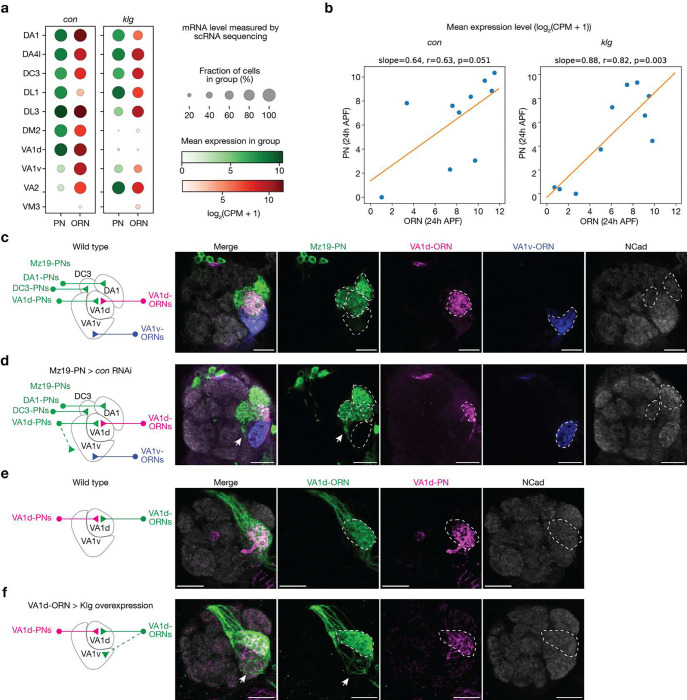
Cell-surface proteins Klg and Con regulate synaptic partner matching in the fly olfactory circuit. Connectin (Con) and Klingon (Klg) are homophilic adhesion molecules and have previously been reported to regulate the wiring of the *Drosophila* visual circuit and neuromuscular system, respectively^17,18^. Here, both the expression pattern and the genetic manipulation results suggest Con and Klg regulate synaptic partner matching of the *Drosophila* olfactory circuits. **a, b,** At around 24 hours after puparium formation (h APF), Con and Klg exhibit matching expression patterns across ORN and PN types based on single-cell RNA sequencing (scRNAseq) data^19,20^. Dot plot (**a**) and scatter plot (**b**) with linear fitting (orange solid line) are shown. Blue dots in (**b**) represent the glomerulus types shown in (**a**). **c**, Maximum projection of optical sections of the same antennal lobes from a wild-type brain. DA1-PNs, DC3PNs and VA1d-PNs (green) are labeled by GFP using *Mz19-GAL4*, VA1d-ORNs (magenta) are labeled by tdTomato using the *Or88a* promoter and VA1v-ORNs (blue) are labeled by rat CD2 using the *Or47b* promoter. The borders of DA1 and DC3 glomeruli are outlined based on the N-cadherin (NCad) staining signal. The VA1v and VA1d glomeruli are outlined according to the ORN signals. The glomerular targeting of these neurons in the wild-type brain is summarized by the schematic on the left. **d**, Same as (**c**), but expressing *con* RNAi in the three Mz19+ PN types. The dendrites of some PNs, likely VA1d-PNs based on anatomical tracing, ectopically target outside glomeruli (arrows). 14 out 14 antennal lobes show similar phenotype using RNAi line 17898 from Vienna *Drosophila* Resource Center and 6 out of 14 antennal lobes show similar phenotype using RNAi line 28967 from Bloomington *Drosophila* Stock Center. **e**, Maximum projection of optical sections of the same antennal lobe from a wild-type brain, with VA1d-ORNs labeled using GFP (green) by GAL4/UAS and VA1d-PNs labeled using tdTomato (magenta) by QF2/QUAS. The border of VA1d glomerulus is outlined based on the N-cadherin (NCad) staining signal. The VA1d-ORN axons and VA1d-PN dendrites match well. **f**, Same as (**e**), but overexpressing Klg in VA1d-ORNs. The axons of some VA1d-ORNs ectopically mistarget to the VA1v glomerulus (arrows).

**Extended Data Fig. 3 |:**
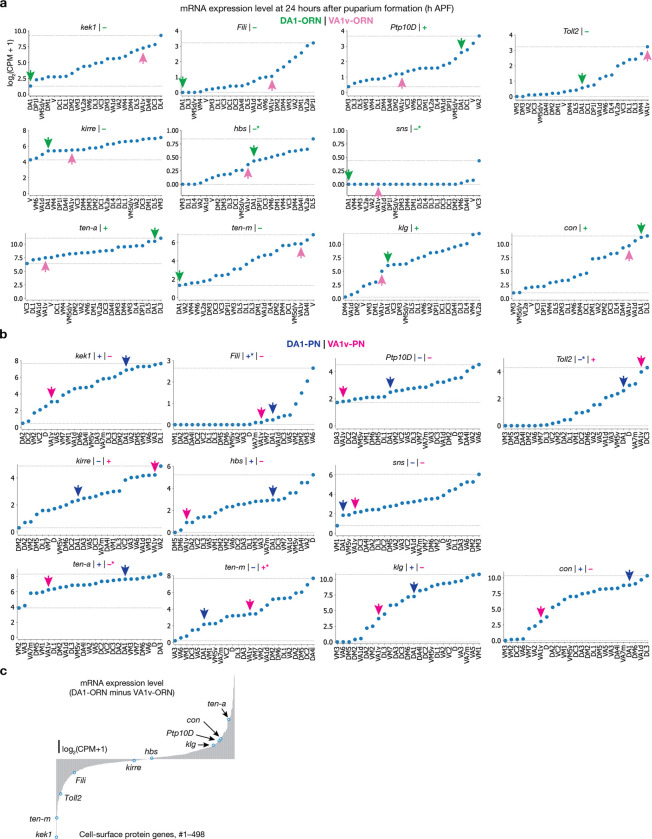
Expression levels of the CSPs in the developing *Drosophila* olfactory system, used in the DA1-ORN rewiring experiments. Here we provide the basis of assigning “+” or “−” for the expression levels of CSPs in Fig. 1d. **a**, mRNA expression levels of the wiring molecules used in the DA1-ORN rewiring experiments. The expression levels are based on the single-cell RNA sequencing (scRNAseq) data^19,20^ and all the ORN types decoded are shown. Plots are generated using data at ~24 hours after puparium formation (h APF) in this and all other panels. In each subplot, the lowest and highest expression levels are indicated by the dashed horizontal lines. The green arrow indicates the data from DA1-ORNs, and the magenta arrow indicates the data from VA1v-ORNs. The ‘+’ or ‘−’ sign indicates the expression level as inferred from the scRNAseq data based on whether the expression level is above (‘+’) or below (‘−’) the midpoint, which is the average of the minimum and maximum value. Since the scRNAseq data are prone to measurement noise and may not accurately reflect protein expression due to post-transcriptional regulation, we corrected expression levels using the protein data and *in vivo* genetic manipulation results in CSPs where additional data were available. * designates places where corrections about the ‘+’ or ‘−’ are made, and the sign showed here is after the correction. Hbs and Sns are considered lowly expressed in both ORN types because of the absolute expression levels of these two mRNAs are very low. The unit of the y axis is log_2_(count per million read + 1) in this and all other panels. **b**, Same as (**a**), but plotting the expression level in all the PN types decoded. The blue arrow indicates the data from DA1-PNs and the magenta arrow indicates the data from VA1v-PNs. Fili is considered highly expressed in DA1-PNs based on the data from a previous study^9^ (in Fig. 3D). Toll2 is considered lowly expressed in DA1-PNs based on the conditional-tag data from the companion study^5^. Ten-a is considered lowly expressed in VA1v-PNs and Ten-m is considered highly expressed in VA1v-PNs based on the antibody staining data from a previous study^6^. **c**, The difference of mRNA expression level of CSPs that are expressed between DA1-ORNs and VA1v-ORNs, with the 10 wiring molecules used in the rewiring experiments indicated. *sns* is not included here since it is lowly expressed in either ORN type.

To achieve rewiring, we considered 7 pairs of CSPs that are likely to signal attractive or repulsive interactions during ORN-PN synaptic partner matching (Fig. 1d). Ten-a and Ten-m are type II transmembrane proteins that each exhibits matching expression patterns across ORN and PN types and mediate homophilic attractive interactions^6^. Connectin (Con) and Klingon (Klg) are homophilic adhesion molecules that participate in the development of *Drosophila* visual circuit and neuromuscular system, respectively^17,18^. Based on single-cell RNA sequencing (scRNAseq) data^19,20^, Con and Klg also exhibit matching expression patterns across ORN and PN types^5^. RNAi-knocking down^21,22^ of *con* and overexpression of Klg caused partial mismatching phenotypes consistent with their promoting homophilic attraction between ORNs and PNs (Extended Data Fig. 2). The remaining seven CSPs form three groups—Kekkon 1(Kek1) with Fish-lips (Fili), Protein tyrosine phosphatas 10D (Ptp10D) with Toll2, and Kin of irre (Kirre) with Hibris (Hbs) and Sticks and stones (Sns)—and signal repulsive interactions between ORNs and PNs^5,9^. Here, we omitted Sns and only considered Kirre and Hbs in our analysis since Sns is lowly expressed in all neurons involved in our rewiring experiments (Extended Data Fig. 3).

The expression level of all CSPs were largely inferred from previously collected scRNAseq data sets during development^19,20^. Since the scRNAseq data are prone to measurement noise and may not accurately reflect protein expression due to post-transcriptional regulation, we corrected our RNA data using protein data and *in vivo* genetic manipulation results in CSPs where additional data were available (Fig. 1d, Extended Data Fig. 3). As summarized in Fig. 1d, developing DA1-ORNs and DA1-PNs in wild type contained attractive interactions from three CSPs (Ten-a, Klg, and Con) but few repulsive interactions from the three repulsive pairs, in accordance with them forming synaptic partners in adults (Fig. 1d, Fig. 2a). By contrast, developing DA1-ORNs and VA1v-PNs contained no attractive interactions from the four attractive CSPs but repulsive interactions from one CSP pair (Ptp10D and Toll2) (Fig. 1b, Extended Data Fig. 3), consistent with them being non-synaptic partners in adults (Fig. 1c, Fig. 2a).

To facilitate rewiring, we utilized three manipulation strategies during development (Fig. 1e–g). First, we increased the repulsion strength between DA1-ORN axons and DA1-PN dendrites (‘*R*+’) to destabilize their interaction. Since repulsive CSPs Kek1, Fili, and Hbs are highly expressed in wild-type DA1-PNs, we overexpressed their interaction partners Fili, Kek1, and Kirre in DA1-ORNs (Fig. 1e). Second, we decreased the strength of repulsion between DA1-ORN axons and VA1v-PN dendrites (‘*R*−’) to stabilize their interaction. Since Ptp10D from DA1-ORNs mediates the repulsive interaction with Toll2 from VA1v-PNs in wild-type flies, we knocked down Ptp10D expression in DA1-ORNs (Fig. 1f). Third, we matched the expression pattern of attractive molecules between DA1-ORN axons and VA1v-PN dendrites (‘Δ*A’*) to stabilize their interactions and at the same time to destabilize the interactions between DA1-ORNs and DA1-PNs. Since the expression pattern of none of the four attractive CSPs between DA1-ORNs and VA1v-PNs match in wild-type flies, we genetically manipulated all four of them independently (Fig. 1g).

## Single-CSP manipulations cause minor rewiring

**Extended Data Fig. 4 |:**
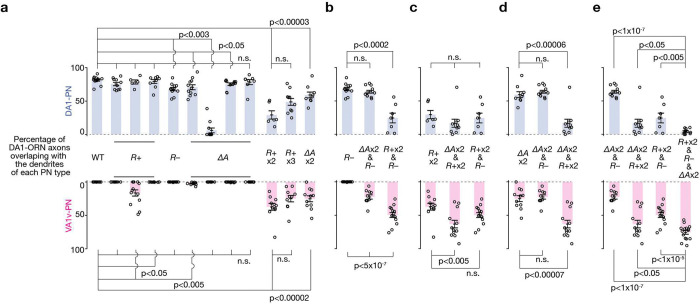
Statistical tests in the DA1-ORNs→VA1v-PNs rewiring. Same plots as in Fig. 2a, but with statistical tests added. Percentage of DA1-ORN axons overlapping with the dendrites of DA1-PNs (top) and VA1v-PNs (bottom). Circles indicate individual antennal lobes; bars indicate the population mean ± s.e.m. ‘*R*+’: Kek1 overexpression (OE), Fili OE, and Kirre OE (left to right). ‘*R*−’: *Ptp10D* RNAi. ‘Δ*A*’: *ten-a* RNAi, Ten-m OE, *klg* RNAi, and *con* RNAi (left to right). ‘*R*+ x2’: Kek1 OE + Fili OE. ‘*R*+ x3’: Kek1 OE + Fili OE + Kirre OE. ‘Δ*A* x2’: *ten-a* RNAi + *con* RNAi. Unpaired t-test is used. **a**, All statistical tests are performed between ‘WT’ and an individual manipulation condition, respectively, except the one test where it is performed between ‘*R*+ x2’ and ‘*R*+ x3’. In all the eight single-CSP manipulations, six—Fili OE, Kirre OE, *Ptp10D* RNAi, *ten-a* RNAi, Ten-m OE, and *klg* RNAi—showed observable yet very mild DA1-ORNs→VA1v-PNs mismatching phenotypes, with some reaching statistical significance while others not (n.s.). **b**, All the tests are performed between ‘*R*−’ and other manipulation conditions, respectively. **c**, All the tests are performed between ‘*R*+ x2’ and other manipulation conditions, respectively. **d**, All the tests are performed between ‘Δ*A* x2’ and other manipulation conditions, respectively. **e**, All the tests are performed between ‘*R*+ x2 & *R*− & Δ*A* x2’ (the DA1-ORN rewired condition) and other manipulation conditions, respectively.

To start, we used the DA1-ORN driver (Extended Data Fig. 1) to overexpress or knockdown different CSPs in DA1-ORNs and to examine their individual effects on synaptic partner matching. All transgenes used in the repulsive interactions were validated in the companion study, where overexpression yielded mismatch phenotypes between ORN axons and PN dendrites across various glomeruli as a positive control and RNAi lines yielded similar phenotypes as mutants^5^. All transgenes used in the attractive interactions were either used in previous studies^6,17,18^ or confirmed via multiple RNAi lines (Extended Data Fig. 2). Across the eight single-CSP manipulations (Fig. 1e–g), six showed observable but subtle DA1-ORNs→VA1v-PNs mismatching phenotypes (Fig. 1h, quantified in Fig. 2a and Extended Data Fig. 4), consistent with the results from the previous manipulation experiments using these CSPs^5,6,9^. In the ‘Ten-m overexpression’ manipulation, there was a major loss of innervation of DA1-ORN axons with DA1-PN dendrites, but none of the mistargeted DA1-ORN axons overlapped with VA1v-PN dendrites. This is consistent with the previous finding that Ten-m-overexpressing DA1-ORN axons most likely mismatch with DL3-PN dendrites^4^. This could be because the CSP profile of DL3-PNs matches with the profile of DA1-ORNs (with Ten-m being overexpressed) better than that of DA1-PNs.

## Combinatorial manipulations enhance rewiring

Next, we simultaneously manipulated the expression of multiple CSPs in DA1-ORNs. We first aimed to find the CSP combination within each of the three manipulation strategies described above (*R*+, *R*−, and Δ*A*; Fig. 1e–g) that can most strongly decrease the overlap between DA1-ORN axons and DA1-PN dendrites (loss of innervation, LoI) and increase the overlap between DA1-ORN axons and VA1v-PN dendrites (gain of innervation, GoI). Overexpression of both Kek1 and Fili (‘*R*+ x2’ in Fig. 2a, b) led to a significant LoI and a significant GoI (Extended Data Fig. 4) compared to overexpressing either alone. This was the largest phenotype we observed among the different combinations of overexpressing repulsive CSPs. For example, overexpressing all three repulsive CSPs (Kek1, Fili, and Kirre, ‘*R*+ x3’ in Fig. 2a, b) improved neither GoI nor LoI compared to ‘*R*+ x2’ (Extended Data Fig. 4). Therefore, we chose overexpressing Kek1 and Fili (‘*R*+ x2’) as the best combination for the strategy of increasing repulsion between DA1-ORNs and DA1-PNs. Similarly, for the strategy of matching the expression pattern of attractive molecules between DA1-ORNs and VA1v-PNs, we found that knocking down Ten-a and Con simultaneously yielded the most significant LoI and GoI (‘Δ*A* x2’ in Fig. 2a, b). We chose knocking down Ptp10D (‘*R*−’ in Fig. 2a, b) as the strategy of decreasing the repulsion of DA1-ORNs and VA1v-PNs since it is the only manipulation available here.

Next, we combined the best options from the three manipulation strategies. When we used two strategies simultaneously (‘Δ*A* x2 & *R*−’, ‘Δ*A* x2 & *R*+ x2’, and ‘*R*+ x2 & *R*−’ in Fig. 2a, b), in most cases, the LoI further decreased and the GoI further increased compared to each strategy alone. For example, in ‘Δ*A* x2 & *R*+ x2’, the LoI was significantly more severe than the LoI in either ‘Δ*A* x2’ or ‘*R*+ x2’, and the GoI in the combined group was also significantly larger than the GoI from each group (Fig. 2a, b, Extended Data Fig. 4). When we combined all three manipulation strategies, nearly all DA1-ORN axons disconnected with DA1-PN dendrites and overlapped with VA1v-PN dendrites (‘*R*+ x2 & *R*− & Δ*A* x2’ in Fig. 2a, c, d). Note that the dendrites of DA1-PNs spread into multiple adjacent glomeruli (inset in Fig. 2c), potentially forming synaptic connections with new ORN partners^10^. Further, DA1-ORN axons only overlapped with part of VA1v-PN dendrites (bottom of Fig. 2c). We confirmed that the non-overlapping part of VA1v-PN dendrites matched with their natural partner VA1v-ORN axons (bottom of Fig. 2c), presumably because we did not genetically manipulate either VA1v-ORNs or VA1v-PNs. Notably, the axons of DA1-ORNs and VA1v-ORNs are segregated in the rewired flies (Fig. 2c), suggesting potential axon-axon repulsive interactions as previously shown in a different context^23^.

**Extended Data Fig. 5 |:**
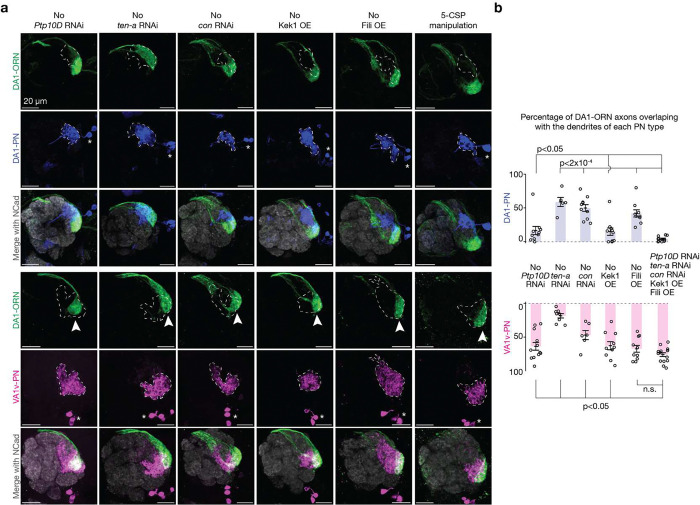
Omitting any one of the five CSP changes reduced the magnitude of DA1-ORN rewiring. **a**, Genetic manipulations are labeled on the top. Maximum z-projections of adult antennal lobes around DA1-ORN axons (green) are shown. Top three rows: DA1-PNs (blue) are co-labeled with borders dashed outlined. Bottom three rows: VA1v-PNs (magenta) are co-labeled with borders dashed outlined. The genetic manipulation condition for the rightmost column contains five genetic changes as listed, same as the rewiring condition as in Fig. 2c. Genetic manipulation conditions for the left five columns are the same as for the rightmost column except missing one manipulation as indicated. Arrowheads indicate the mismatch of DA1-ORN axons with VA1v-PN dendrites; Scale bar = 20 μm; * designates PN cell bodies. **b**, Percentage of DA1-ORN axons overlapping with the dendrites of DA1-PNs (top) and VA1v-PNs (bottom). Same genetic manipulation conditions as (**a**). Circles indicate individual antennal lobes; bars indicate the population mean ± s.e.m. All the tests are performed between the data from the rightmost column and the data from other columns, respectively. Unpaired t-test is used. n.s., not significant.

**Extended Data Fig. 6 |:**
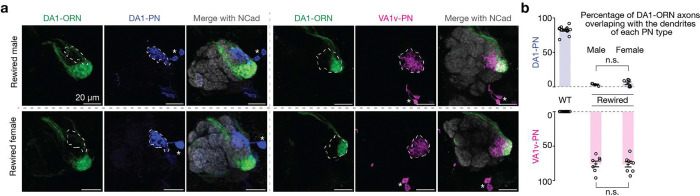
Males and females exhibit similar DA1-ORN→VA1v-PN rewiring. **a**, Maximum z-projections of adult antennal lobes around DA1-ORN axons (green) are shown. Top row: rewired male; bottom row: rewired female. Left three columns: DA1-PNs (blue) are co-labeled with borders dashed outlined; right three columns: VA1v-PNs (magenta) are co-labeled with borders dashed outlined. The genetic manipulation condition is the same as the rewiring condition as in Fig. 2c. Scale bar = 20 μm; * designates PN cell bodies. **b**, Percentage of DA1-ORN axons overlapping with the dendrites of DA1-PNs (top) and VA1v-PNs (bottom). Circles indicate individual antennal lobes; bars indicate the population mean ± s.e.m. Unpaired t-tests are performed.

In this final rewiring experiment (referred hereafter as DA1-ORN rewired flies), the expression levels of five CSPs were changed in DA1-ORNs (Kek1, Fili, Ptp10D, Ten-a, and Con; Fig. 2c). When any one of the five CSP changes was omitted, the rewiring was less complete (Extended Data Fig. 5). Despite the fly DA1 glomerulus being sexually dimorphic in size^24,25^, the rewiring of DA1-ORN axons to VA1v-PN dendrites showed similar levels of change in male and female flies (Extended Data Fig. 6).

## Rewiring alters VA1v-PN odor response

**Fig. 3 |:**
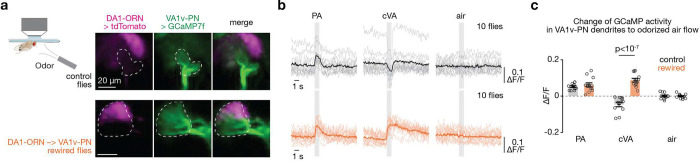
VA1v-PNs in DA1-ORN rewired flies respond to both VA1v- and DA1-specific odors. **a**, Imaging neural activity in a plate-tethered fly with odorized air flow delivered to the fly antennae. Images of tdTomato signal in DA1-ORN axons and GCaMP7f signal in VA1v-PN dendrites are shown from a control fly (top) and a DA1-ORN rewired fly (bottom). Images are averaged across the entire recording. The VA1v glomerulus is outlined according to GCaMP7f signal. Scale bar = 20 μm. Note that the imaging angle here is from dorsal to ventral whereas all the other staining images are from anterior to posterior. **b**, Averaged GCaMP7f activity in VA1v-PN dendrites in response to odorized air flows, measured by fluorescence intensity change over baseline (ΔF/F). Top: control flies. Bottom: DA1-ORN rewired flies. The grey vertical stripes indicate odorized air flows (1s each). Light-colored traces indicate the means of individual flies; dark-colored traces indicate the population mean. In wild-type flies, palmitoleic acid (PA) specifically activates VA1v-ORNs^12^ and 11-*cis*-vaccenyl acetate (cVA) specifically activates DA1-ORNs^13,28,29^. **c**, Change of GCaMP7f activity in VA1v-PN dendrites to odorized air flows. The change of activity is calculated by subtracting the average GCaMP7f activity in the 0.5 s before the onset of odor delivery from that in the last 0.5 s of odorized airflow. Circles indicate the means of individual flies; bars indicate the population mean ± s.e.m. Unpaired t-test is used.

To examine whether the anatomical rewiring of DA1-ORN axons to VA1v-PN dendrites is accompanied with the formation of functional synaptic connections, we measured the neural response of VA1v-PN dendrites to VA1v- or DA1-specific odors in tethered flies (Fig. 3a). All ORN-PN connections are excitatory and use the same cholinergic neurotransmitter system, including DA1 and VA1v^26^. We used the LexA/LexAop system to express GCaMP7f in VA1v-PNs and measured intracellular Ca^2+^ concentrations via two-photon excitation of GCaMP7f^27^ as a proxy for neural activity. We simultaneously expressed and co-imaged tdTomato in DA1-ORNs with GCaMP7f to confirm the occurrence of DA1-ORN rewiring in these flies (Fig. 3a).

We next tested odor responses of VA1v-PN dendrites. 11-*cis*-vaccenyl acetate (cVA) is a pheromone that specifically activates DA1-ORNs in the fly antennal lobe^13,28,29^. Palmitoleic acid (PA) is a fly cuticular pheromone that specifically activates VA1v-ORNs in the fly antennal lobe^12^. In wild-type flies, we found that the dendrites of VA1v-PNs increased response to PA and decreased response to cVA (Fig. 3b, c). The inhibitory response of VA1v-PNs to cVA in wild-type flies is consistent with the previously described lateral inhibition from local interneurons in the fly olfactory circuit^30,31^. In the rewired flies, however, both PA and cVA activated VA1v-PNs (Fig. 3b, c), supporting functional synaptic connections between DA1-ORN axons and VA1v-PN dendrites. We cannot rule out the possibility that altered connectivity of local interneurons, which exhibit diverse anatomical patterns^32,33^, also contributes to the altered odor response.

## Rewiring promotes male-male courtship

Would DA1-ORNs→VA1v-PNs rewiring lead to any behavioral change in flies? In *Drosophila melanogaster*, cVA is only produced in males and acts through the Or67d odorant receptor expressed in DA1-ORNs to inhibit the courtship of males towards other males or recently mated females^13^ (cVA can be transferred to females during copulation^34^). The pheromone PA, on the other hand, has been shown to promote courtship in males through the Or47b odorant receptor expressed in VA1v-ORNs^12^. Therefore, in the rewired flies, an odor (cVA) that inhibits courtship in wild-type males is rerouted to a pathway (VA1v) that promotes courtship, potentially boosting male-male courtship.

**Extended Data Fig. 7 |:**
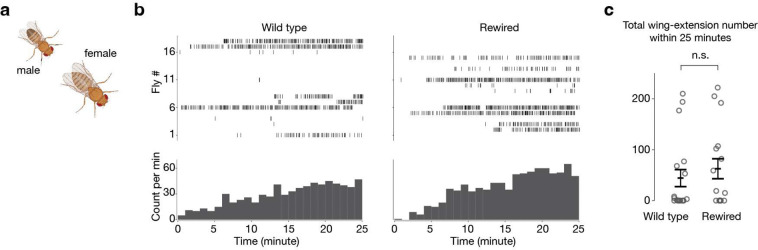
Wild-type and DA1-ORN→VA1v-PN rewired males exhibit similar courtship activity towards virgin females. **a**, Courtship assay where one male and one virgin female are introduced in the same behavioral chamber to monitor the courtship activity from the male towards the female. The chamber diameter is 2 cm. **b**, Unilateral-wing-extension rasters (top) and extension count per minute (1-min bins, bottom). Left: wild-type males; right: DA1-ORN rewired males. **c**, Total wing-extension number during the 25-minute recordings. Circles indicate individual flies; bars indicate the population mean ± s.e.m. Wilcoxon signed-rank test is used given the non-normal distribution of the data points.

**Fig. 4 |:**
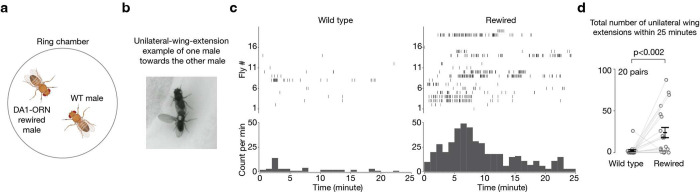
Male flies with DA1-ORNs rewired show elevated courtship activity towards other males. **a**, Courtship assay where one wild-type male and one DA1-ORN rewired male are introduced in the same behavioral chamber to monitor their courtship activity towards each other. The chamber diameter is 2 cm. **b**, Example frame of unilateral wing extension from a DA1-ORN rewired male (white dot on the thorax) towards a wild-type male. **c**, Rasters of unilateral wing extensions (top) and extension count per minute (1-min bins, bottom). Left: wild-type males; right: DA1-ORN rewired males. The same fly number in the left and right panels are the fly pair from the same experiment. **d**, Total unilateral-wing-extension number within 25-minute recordings. Circles indicate individual flies; bars indicate the population mean ± s.e.m. Wilcoxon signed-rank test is used given the non-normal distribution of the data points.

To test this, we introduced two virgin males—one wild type and one with DA1-ORN rewired—into the same behavioral chamber (Fig. 4a). We then recorded video for 25 minutes and analyzed from both males the unilateral-wing-extension events (Fig. 4b, Videos 1, 2), a typical male courtship behavior during which males vibrate one of their wings to produce courtship song^35^. We found that the rewired males exhibited unilateral wing extensions towards their wild-type partner males significantly more frequently than the other way around (Fig. 4c, d). In a separate experiment, we introduced one male—either wild type or rewired—with a virgin female into the behavioral chamber. We did not observe any detectable differences in the courtship activity towards virgin females between wild-type and rewired males (Extended Data Fig. 7). This is consistent with our working model since a virgin female does not emit cVA, and connections between VA1v-ORNs and VA1v-PNs in rewired flies remained intact as assayed anatomically (Fig. 2c) and physiologically (Fig. 3b, c). Finally, when five virgin rewired males were introduced into the same behavioral chamber, they exhibited vigorous chasing and courtship activities, sometimes forming a courtship chain in which a male attempted to court the male in front of him while being courted by another male behind him (Video 3).

## Generalization to other glomeruli

**Fig. 5 |:**
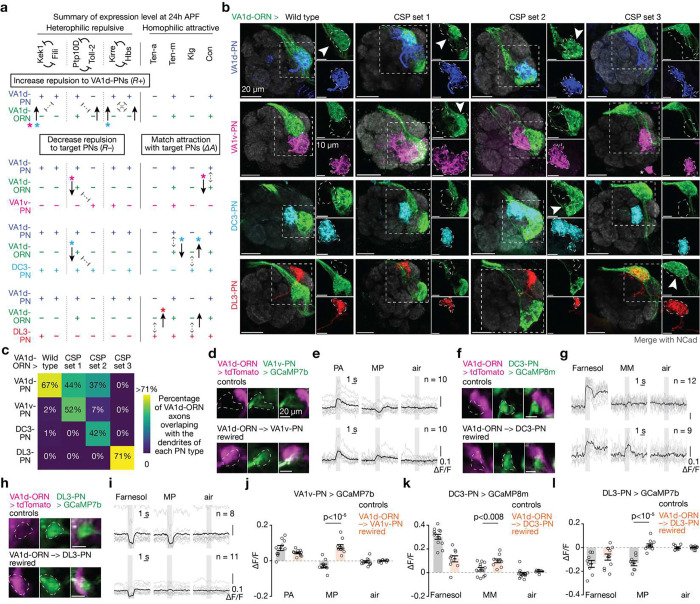
Rewiring VA1d-ORNs with distinct PN partners following the same manipulation strategies. **a**, Summary of expression levels of the ten CSPs and the three manipulation strategies considered in the experiments of rewiring VA1d-ORNs (green) away from VA1d-PNs (blue) and towards VA1v-PNs (magenta), DC3-PNs (cyan), or DL3-PNs (red), respectively. Same color code here and in (**b**). Nomenclature same as Fig. 1d–g. Available protein expression data are in Extended Data Fig. 8. * indicates the genetic manipulations used in the final switches of VA1d-ORNs to different PN types. See main text for more details. **b**, Maximum z-projections of adult antennal lobes around VA1d-ORN axons in wild type and all the three rewired experiments. In each row, VA1d-ORNs are co-labeled with one PN type according to labels on the left. In each column, the same set of genetic manipulations is used. CSP set 1 = Kek1 overexpression (OE) + *Ptp10D* RNAi + *con* RNAi + *sema-2b* RNAi; CSP set 2 = Kek1 OE + Kirre OE + *Ptp10D* RNAi + *ten-m* RNAi + Klg OE; CSP set 3 = Ten-a OE + *sema-2b* RNAi. *sema-2b* RNAi is used for rerouting the VA1d-ORN trajectory to better match the dendrites of VA1v-PNs and DL3-PNs. See main text and Extended Data Fig. 9 for detail. Borders of the dendrites of each PN type are dash-outlined. Arrowheads indicate the overlap of VA1d-ORN axons with the dendrites of specific PN types. Insets are zoom-ins from the dashed squares to their left. Scale bar = 20 μm in large images. Scale bar = 10 μm for insets. * designates PN cell bodies. **c**, Percentage of VA1d-ORN axons overlapping with the dendrites of each PN type (indicated on the left) in wild type and the three rewired conditions (indicated on the top). n ≥ 6 for all the conditions. See Extended Data Fig. 9 for detail. **d**, Images of tdTomato signal in VA1d-ORN dendrites and GCaMP7f signal in VA1v-PN dendrites are shown from a control fly (top) and a VA1d-ORN**→**VA1v-PN fly (bottom). Images are averaged across the entire recording. The VA1v glomerulus is outlined according to GCaMP7f signal. Scale bar = 20 μm. Same imaging set up as in Fig. 3. **e**, Averaged GCaMP7f activity in VA1v-PN dendrites in response to odorized air flows. Top: control flies. Bottom: VA1d-ORN**→**VA1v-PN flies. The grey squares indicate odor deliveries (1s each). Grey lines indicate the means of individual flies; black lines indicate the population mean. In wild-type flies, palmitoleic acid (PA) specifically activate VA1v-ORNs^12^ and methyl palmitate (MP) mainly activate VA1d-ORNs^28^. **f, g,** Same as (**d, e**), but examining rewiring of VA1d-ORNs to DC3-PNs instead of VA1v-PNs. GCaMP8m is used instead of GCaMP7f. In wild-type flies, farnesol mainly activates DC3-ORNs^36^. Methyl myristate (MM) specifically activate VA1d-ORNs and VA1v-ORNs^12^ and is used here instead of MP since we observed positive response of DC3-PNs to MP in wild type (our unpublished observation). **h, i**, Same as (**d, e**), but examining rewiring of VA1d-ORNs to DL3-PNs instead of VA1v-PNs. Note that farnesol is used as a control since DL3-specific odorants remain unknown. In the control fly image, VA1d-ORN signal is absent because VA1d-ORN axons and DL3-PN dendrites occupy different z positions from the current imaging perspective. **j–l**, Change of GCaMP activity in dendrites of VA1v-PNs (**j**), DC3-PNs (**k**), and DL3-PNs (**l**) to odorized air flow. The change of activity is calculated by subtracting the average GCaMP activity in the 0.5 s before the onset of odor delivery from the average GCaMP activity in the last 0.5 s of odorized air flow. Circles indicate the means of individual flies; bars indicate the population mean ± s.e.m. Same number of flies as shown in panels e, g, and i, respectively. Unpaired t-test is used.

Do the same set of CSPs and wiring strategies also apply to the ORN-PN synaptic partner matching in other glomeruli? We tested this by attempting to rewire the axons of another ORN type, VA1d-ORNs, to the dendrites of PNs targeting three distinct neighboring glomeruli: VA1v, DC3, and DL3 (Fig. 5).

We used a genetic driver that specifically labels VA1d-ORNs across developmental stages using split-GAL4^4^, and simultaneously labeled the dendrites of either VA1d-PNs, VA1v-PNs, DC3-PNs, or DL3-PNs in the same adult brain using the orthogonal LexA/LexAop^16^ or QF/QUAS^15^ systems (Fig. 5b). In wild-type flies, VA1d-ORN axons almost exclusively overlapped with the dendrites of VA1d-PNs and showed minimal overlap with the dendrites of other PN types (Fig. 5b, c). The goal of rewiring is to switch the axons of VA1d-ORNs to match with the dendrites of each of the three other PN types in individual experiments.

**Extended Data Fig. 8 |:**
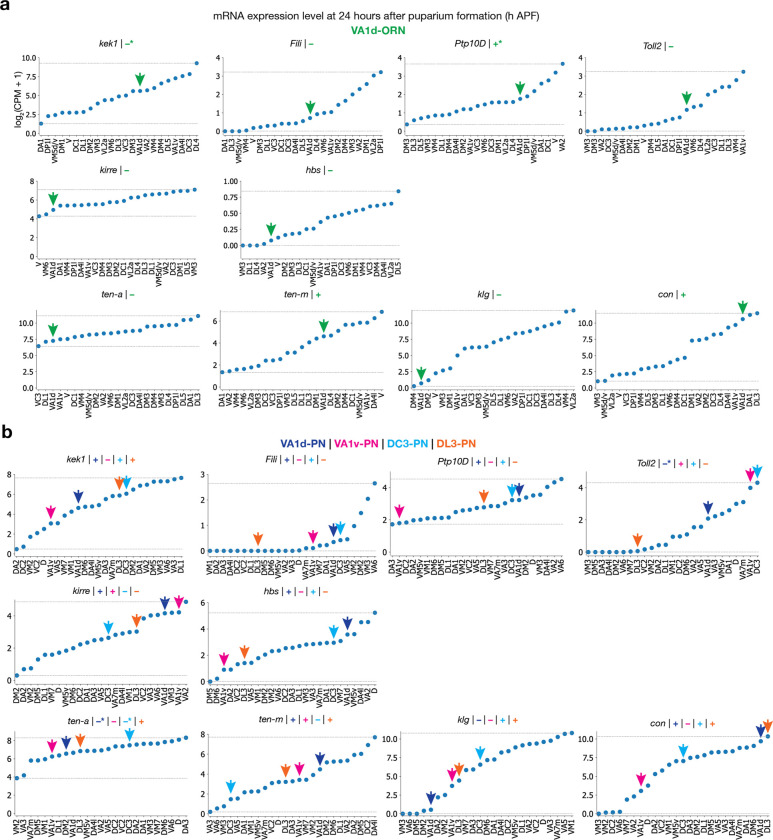
Expression levels of the ten CSPs in the VA1d-ORN rewiring. Here we provide the basis of assigning “+” or “−” for the expression levels of CSPs in Fig. 5a. **a**, mRNA expression levels of the 10 CSPs used in the VA1d-ORN rewiring experiments. The expression levels are based on the single-cell RNA sequencing (scRNAseq) data^19,20^ and all the ORN types decoded are shown. Plots are generated using data at 24 hours after puparium formation (h APF) in this and all other panels. In each subplot, the lowest and highest expression levels are indicated by the dashed horizontal lines. The green arrow indicates the data from VA1d-ORNs. The ‘+’ or ‘−’ sign indicates the expression level as inferred from the scRNAseq data based on whether the expression level is above (‘+’) or below (‘−’) the midpoint, which is the average of the minimum and maximum value. Since the scRNAseq data are prone to measurement noise and may not accurately reflect protein expression due to post-transcriptional regulation, we corrected the RNA data using the protein data and the in vivo genetic manipulation results in CSPs where additional data were available. * designates places where corrections are made, and the sign showed here is after the correction. Kek1 is considered lowly expressed based on the conditional-tag data from the companion study^5^. Ptp10D is considered highly expressed based on the conditional-tag data as well as the knockout experiments from the companion study^5^. The unit of the y axis is log_2_(count per million read + 1). **b**, Same as (**a**), but plotting the expression level in all the PN types decoded. The blue arrow indicates the data from DA1-PNs, the magenta arrow indicates the data from VA1v-PNs, light blue arrow indicates the data from DC3-PNs, and the orange arrow indicates the data from DL3-PNs. Toll2 is considered lowly expressed in VA1d-PNs based on the conditional-tag data from the companion study^5^. Ten-a is considered lowly expressed in VA1d- and DL3-PNs based on the antibody staining data from a previous study^6^.

Based on the same ten CSPs described above, during the development of wild-type flies, VA1d-ORN axons and VA1d-PN dendrites form two attractive interactions (via Ten-m and Con) and show few repulsive interactions (first row in Fig. 5a, Extended Data Fig. 8). For the first manipulation strategy that aims to increase the strength of repulsion between VA1d-ORNs and VA1d-PNs, we could overexpress repulsive CSPs Kek1, Toll2, Kirre, or Hbs in all three rewiring attempts (first row in Fig. 5a). For the second manipulation strategy that aims to decrease the strength of repulsion between VA1d-ORNs and other PN types, we sought to knock down Ptp10D in two of the three switch attempts and do nothing in the switch attempt to DL3-PNs since DL3-PNs do not exhibit any repulsive interactions with VA1d-ORNs from these three repulsive pairs (bottom three rows in Fig. 5a, left half). For the third manipulation strategy that aims to match the expression pattern of attractive molecules between VA1d-ORNs and other PN types, we could overexpress or knock down the expression of these four attractive CSPs accordingly (bottom three rows in Fig. 5a, right half).

**Extended Data Fig. 9 |:**
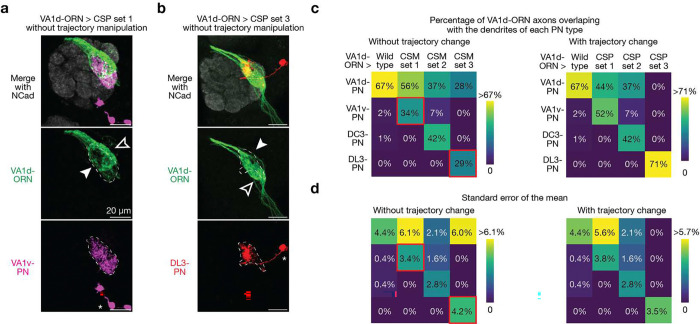
Trajectory manipulation of VA1d-ORN axons is necessary for rewiring with the dendrites of VA1v- and DL3-PNs. **a**, Maximum z-projections of adult antennal lobes around VA1d-ORN axons in the rewiring experiments without trajectory manipulation of VA1d-ORN axons. Genetic manipulations = Kek1 overexpression (OE) + *Ptp10D* RNAi + *con* RNAi. VA1v-PNs (magenta) are co-labeled with borders dashed outlined. The solid arrowhead indicates the mismatch of VA1d-ORN axons with VA1v-PN dendrites; the open arrowhead indicates the part of VA1d-ORN axons that do not match with VA1v-PN dendrites. Scale bar = 20 μm; * designates PN cell bodies. **b**, Same as (**a**), but for the rewiring experiment of VA1d-ORN axons to DL3-PN dendrites. Genetic manipulations = Ten-a OE. DL3-PNs (red) are co-labeled with borders dashed outlined. The solid arrowhead indicates the mismatch of VA1d-ORN axons with DL3-PN dendrites; the open arrowhead indicates the part of VA1d-ORN axons that do not match with DL3-PN dendrites. **c**, Percentage of VA1d-ORN axons overlapping with the dendrites of each PN type (indicated on the left) in wild type and the three rewired conditions without (left) and with (right) trajectory manipulations. The right matrix is repeated from Fig. 5c for easy comparison. The two red boxes in the left matrix indicate the genetic manipulation conditions showed in (**a**) and (**b**), respectively. Note that in these two boxes, the ratios of VA1dORN axons rewired to the target PNs are less compared to the conditions with trajectory manipulations, suggesting that trajectory manipulation of VA1d-ORN axons is necessary in the rewiring to the dendrites of VA1v- and DL3-PNs. n ≥ 6 for all the conditions. **d**, Same as (**c**), but plotting the s.e.m. instead of the population mean.

By combining the different specific manipulations described above, we were able to rewire more than half of VA1d-ORN axons to match with the dendrites of either VA1v-PNs or DC3-PNs in two separate experiments, and to rewire almost all VA1d-ORN axons to match with DL3-PN dendrites in a third experiment (Fig. 5b, c). In all three rewiring experiments, the part of VA1d-ORN axons that did not match with the dendrites of target PNs remained matching with dendrites of their natural partner VA1d-PNs (Fig. 5b, c). Note that in the rewiring to VA1v-PNs and DL3-PNs, we also included an additional manipulation, Sema-2b knockdown. This is because VA1v-ORN and DL3-ORN axons take a more dorsolateral trajectory than VA1d-ORNs when they sweep through the antennal lobe surface^11^. Since single ORN axons mainly searches in the vicinity of their trajectory^10^, we included Sema-2b knockdown to shift the axons of VA1d-ORNs more dorsolaterally^11^ so their trajectories could be closer to the dendrites of VA1v-PNs and DL3-PNs. Consistently, when all the manipulations remained the same but leaving out the Sema-2b knockdown, there was less matching between VA1d-ORN axons and VA1v- or DL3-PN dendrites (Extended Data Fig. 9).

To test whether the anatomical rewiring of VA1d-ORNs described above lead to the formation of functional synaptic connections, we examined in rewired flies if the different PN types gain responses to VA1d-ORN specific odors^12,28^. Using the same setup as in Fig. 3a, we measured the neural response of VA1v-, DC3-, and DL3-PNs, separately, via two-photon excitation of GCaMP variants expressed using the LexA/LexAop or QF/QUAS system in these PNs (Fig. 5d–i). We also co-expressed tdTomato in VA1d-ORNs to confirm the anatomical switch in these flies. In all three rewiring experiments, the dendrites of target PNs gained response to VA1d-ORN-specific odors compared to in wild-type flies (Fig. 5d–l). Altogether, these results demonstrate that the three genetic strategies for altering the cell-surface combinatorial code are generalizable for selecting synaptic partners in the fly olfactory circuit.

## Discussion

Here, we demonstrated that the fly olfactory circuit can be largely, in some cases completely, rewired when 2-to-5 cell-surface proteins (CSPs) are changed in a single ORN type (Figs. 1, 2, and 5). This occurs even though dozens of CSPs are differentially expressed between different ORN types during the synaptic partner matching period (Extended Data Fig. 3). The rewiring expanded the physiological response to odors in downstream PNs (Figs. 3 and 5) and altered the courtship behavior in one case (Fig. 4).

The CSP combinatorial code for rewiring should be closely related, if not identical, to the CSP code used during natural wiring. To illustrate, consider the rewiring of DA1-ORNs→VA1v-PNs. First, the five CSPs involved in the rewiring are differentially expressed between DA1-ORNs and VA1v-ORNs (Extended Data Fig. 3). The directions of gene expression manipulation—whether up- or down-regulation—match the discrepancy between these two ORN types. Second, both loss-of-function and gain-of-function manipulations in most of the five CSPs alone significantly decreased the matching of DA1-ORN axons with DA1-PN dendrites or caused mismatch of DA1-ORN axons with VA1v-PN dendrites (Extended Data Fig. 4), supporting the involvement of these CSPs in distinguishing the wiring specificity of DA1-ORNs and VA1v-ORNs naturally. Finally, rewiring leads to a gain-of-function at both the physiological and behavioral levels, suggesting its potential usage in an evolutionary context.

The results that the rewiring could be successful despite our lack of precise control over the level and timing of the CSP manipulations suggests that the combination of key CSPs is more critical than the exact levels and timing of their expression. This is consistent with the general notion that many biological systems are robust in their tolerance to variations in expression levels. The precision of rewiring may be further improved if we could better control our genetic manipulations in level and timing, and by manipulating additional CSPs that we may have missed (e.g., in the case of VA1d-ORNs→DA1-PNs and VA1d-ORNs→DC3-PNs in Fig. 5). Finally, our results show that CSPs of different families^5,6,17,18^—those containing immunoglobulin-like domain (Klg, Kirre, Hbs, Kek1), leucine-rich repeats (Con, Fili, Kek1, Toll2), fibronectin III domains (Ptp10D, Hbs), and teneurin (Ten-a, Ten-m)—work together in different combinations for synaptic partner matching at different glomeruli. These data suggest the system is flexible in the specific CSPs used in synaptic partner matching as long as they execute a set of strategies: matching attractive CSPs between partners, avoiding repulsive CSPs between partners, and displaying repulsive CSPs between non-partners.

## Methods

### Fly husbandry and stocks

Flies were reared on a standard cornmeal medium at 25°C under a 12-hour light and 12-hour dark cycle. To enhance transgene expression levels, flies from all genetic perturbation experiments, including control groups, were shifted to 29°C shortly before puparium formation. Detailed genotypes for each experiment are listed in Extended Data Table 1.

### Molecular cloning and generation of transgenic flies

To generate QF2 lines, we used *pENTR/D-TOPO* vectors with different enhancer insertions (gifts from G. Rubin lab) as entry vectors for Gateway cloning into the *pBPQF2Uw* vector using LR Clonase II Enzyme mix (Invitrogen, 11791020). *pBPQF2Uw* was made using NEBuilder HiFi DNA assembly master mix (New England Biolabs) to replace the GAL4 on *pBPGAL4.2Uw-2* vector (Addgene #26227) with QF2 from *pBPGUw-HACK-QF2* (Addgene #80276). The resulting constructs were sequence-verified and inserted into JK22C landing sites by Bestgene. *pGP-5XQUAS-IVS-Syn21-jGCaMP8m-p10* was made using NEBuilder HiFi DNA assembly master mix (New England Biolabs) to replace the 20XUAS on *pGP-20XUAS-IVS-Syn21-jGCaMP8m-p10* vector (Addgene #162387) with 5XQUAS from pQUAST (Addgene #24349). *UAS-Fili-Flag* was made using NEBuilder HiFi DNA assembly master mix (New England Biolabs) to remove the V5 tag on *UAS-SP-V5-Fili-Flag*^9^. Plasmids were injected to embryos at Bestgene. Genetic labeling with these drivers is unlikely to disrupt normal development, as a previous study showed that drivers with improved translation efficiency could elevate GFP expression by 20 fold with no apparent effect on neuronal morphology^37^.

### Immunostaining

The procedures used for fly dissection, brain fixation, and immunostaining were described previously^10^. For primary antibodies, we used rat anti-DNcad (1:30, from DSHB, RRID # AB_528121), chicken anti-GFP (1:1000, from Aves Labs, RRID # AB_10000240), rabbit anti-DsRed (1:500, from Takara Bio, RRID # AB_10013483), and mouse anti-rat CD2 (1:200; OX-34, Bio-Rad).

### Confocal imaging

Immunostained brains were imaged using a laser-scanning confocal microscope (Zeiss LSM 780). Images of antennal lobes were taken as confocal stacks with 1-mm-thick sections. Representative single sections were shown to illustrate the arborization features of ORN axons and PN dendrites, with brightness adjustment, contrast adjustment, and image cropping done in ImageJ.

### Calculating the percentage of ORN axons matching with PN dendrites

PN dendritic pixels and ORN axonal pixels were defined by first smoothening the image using ‘gaussian blur’ (radius = 2 pixels) and then thresholding the image based on the algorithm ‘Otsu’ in Fiji. We found that this algorithm could efficiently separate the neurons of interest from the background. Irrelevant signals (such as the PN axons, cell bodies, or autofluorescence) that still persisted after the above operations were manually masked out in the analysis. A portion of ORN axons was considered as matching with PN dendrites if they have overlapping pixels on a single *z*-plane in the image.

### Ca^2+^ imaging and data analysis

#### Odor stimuli delivery.

10 μL of odorant palmitoleic acid (Thermo Fisher, 376910010) and 11-*cis*-vaccenyl acetate (cVA; Cayman Chemical, 10010101) was applied to filter paper (Amazon, B07M6QJ2JX) inserted inside a 1 ml pipette tip. The pipette tip was left aside for at least half an hour before being positioned approximately 5 mm away from the fly antenna. Close positioning of palmitoleic acid and cVA is necessary because both odorants are large pheromone molecules with relatively low volatility. This method has also been utilized in other studies^12^. Other odorants, such as methyl palmitate (Thermo Fisher, L05509.36), methyl myristate (Thermo Fisher, 165015000), and farnesol (Thermo Fisher, 119121000), were stored in a small glass bottle and delivered to the fly antenna via tubing with a 10% dilution in heavy mineral oil on the day of experiments. A constant stream of charcoal-filtered air (1 L/min) was directed towards the fly, switching to odorant-containing air for 1 second as the odor stimulus before returning to the air stream. A pulse of charcoalfiltered air served as a negative control. Odorants, including the control pulse, were interleaved with at least 15-second intervals. Each odorant was delivered two to three times per recording, with the delivery sequence shuffled within each cycle. As described previously^38^, we glued flies to a custom stage. Dissection and imaging protocols also followed a previous study^38^.

#### Data acquisition and alignment.

We used a two-photon microscope with a moveable objective (Ultima IV, Bruker). The two-photon laser (Chameleon Ultra II Ti:Sapphire, Coherent) was tuned to 925 nm in all of the imaging experiments. We used a ×16/0.8 NA objective (Nikon) for all imaging experiments. The laser intensity at the sample was 15–30 mW. A 575-nm dichroic split the emission light. A 490–560-nm bandpass filter (Chroma) was used for the green channel and a 590–650-nm bandpass filter (Chroma) was used for the red channel. We recorded all imaging data using single z plane, at a rate of 9–13 Hz. We perfused the brain with extracellular saline composed of (in mM) 103 NaCl, 3 KCl, 5 N-Tris(hydroxymethyl) methyl-2-aminoethanesulfonic acid (TES), 10 trehalose, 10 glucose, 2 sucrose, 26 NaHCO_3_, 1 NaH_2_PO_4_, 1.5 CaCl_2_, 4 MgCl_2_. All data were digitized by a Digidata 1550b (Molecular Devices) at 10 kHz, except for the two-photon images, which were acquired using PrairieView (Bruker) at varying frequencies and saved as tiff files for later analysis. We used the frame triggers associated with our imaging frames (from Prairie View), recorded on Digidata 1550b, to carefully align odorant delivery with [Ca^2+^] imaging measurements.

#### Image registration.

The image stacks were motion-corrected using non-rigid motion correction (NoRMCorre^39^) and then manually validated to check for motion artifacts.

#### Defining regions of interest.

To analyze Ca^2+^ imaging data, we defined regions of interest (ROIs) in Fiji and Python for GCaMP signals from projection neurons in one side of the brain, or both sides when the PN dendritic signals are available. We treated the entire PN dendrite from one side of the brain as one ROI.

#### Calculating fluorescence intensities.

We used ROIs, defined above, as the unit for calculating fluorescent intensities (see above). For each ROI, we calculated the mean pixel value at each time point and then used the method ∆F/F0 to calculate, where F0 is the mean of the lowest 5% of raw fluorescence values in a given ROI over time and ∆F is F – F0.

### Courtship assay

Flies were collected shortly after eclosion. Male flies were housed individually, while female flies (Canton-S) were housed in groups of approximately 10. All males tested in the experiments were 4–7 days old and had not mated. All females used as courtship targets were 3–5 days old virgins. All flies were either w+ or carried more than three mini-white markers from the transgenes they possess. In single-pair courtship assays, two males or one male and one female were introduced into a custom-made courtship chamber with a diameter of 2 cm. In the courtship chain assay, five DA1-ORNs→VA1v-PNs males were introduced into a custom-made courtship chamber with a diameter of 5 cm. Courtship experiments were conducted under faint white light to lower baseline courtship activity, as vision has been widely shown to influence the vigor of fly courtship. Before being placed into the courtship chamber, flies were briefly grouped in a tube and anesthetized on ice for less than 10 seconds. Once placed into the chamber, most flies were able to move immediately but did not fly away. Fly behavior was recorded for >25 minutes with a video camera at 13 frames per second and the first 25 minutes were quantified. In the single-pair male-male courtship assay, a control male and a rewired male were age-matched, and one of them was marked with an oil paint marker (Sharpie) on their thorax at least one day before the experiment. The paint was alternated between control and rewired males.

## Extended Data

**Extended Data Table 1 |:** Summary of genotypes used in each experiment, arranged according to figure panels.

Figure and panel	description	Fly genotype		
		x chromosome	2nd chromosome	3rd chromosome
Figure 1				
b	wild type DA1 -ORN and DA1-PN	*UAS-Dcr2 (BDSC 24646), UAS-mCD8-GFP (BDSC 24648) / w or y*	*R24A10-QF2 (this work), QUAS-mtdTomato-HA (BDSC 30004) / +*	*R78H05-p65.AD (BDSC 601815), R22E04-GAL4.DBD (BDSC 68303) / +*
c	wild type DA1 -ORN and VA1v-PN	*UAS-Dcr2 / w or y*	*VT003280-LexA (BDSC 94678), lexAop-rCD2::RFP-p10.UAS-mCD8::GFP-p10 (BDSC 67093) / +*	*R78H05-p65.AD, R22E04-GAL4.DBD / +*
h	Kek1 overexpression with DA1-PN colabeling	*UAS-Dcr2, UAS-mCD8-GFP / w or y*	*R24A10-QF2, QUAS-mtdTomato-HA / IAS Kek1 (BDSC 43665)*	*R78H05-p65.AD, R22E04-GAL4.DBD / +*
	Fili overexpression with DA1-PN colabeling	*UAS-Dcr2, UAS-mCD8-GFP / w or y*	*R24A10-QF2, QUAS-mtdTomato-HA / +*	*R78H05-p65.AD, R22E04-GAL4.DBD / UAS-Fili (this work)*
	Kirre overexpression with DA1-PN colabeling	*UAS-Dcr2, UAS-mCD8-GFP / UAS-Kirre (BDSC 26609)*	*R24A10-QF2, QUAS-mtdTomato-HA / +*	*R78H05-p65.AD, R22E04-GAL4.DBD / +*
	Ptp10D RNAi with DA1-PN colabeling	*UAS-Dcr2, UAS-mCD8-GFP / w or y*	*R24A10-QF2, QUAS-mtdTomato-HA / +*	*R78H05-p65.AD, R22E04-GAL4.DBD / UAS-Ptp10D-RNAi (VDRC 8010)*
	*ten-a* RNAi with DA 1 - PN colabeling	*UAS-Dcr2, UAS-mCD8-GFP / w or y*	*R24A10-QF2, QUAS-mtdTomato-HA / +*	*R78H05-p65.AD, R22E04-GAL4.DBD / UAS-Ten-a-RNAi (BDSC 29439)*
	Ten-m overexpression with DA1-PN colabeling	*UAS-Dcr2, UAS-mCD8-GFP / w or y*	*R24A10-QF2, QUAS-mtdTomato-HA / +*	*R78H05-p65.AD, R22E04-GAL4.DBD / UAS-Ten-m (BDSC 41567)*
	*klg* RNAi with DA1-PN colabeling	*UAS-Dcr2, UAS-mCD8-GFP / w or y*	*R24A10-QF2, QUAS-mtdTomato-HA / +*	*R78H05-p65.AD, R22E04-GAL4.DBD / UAS-klg-RNAi (BDSC 28746)*
	*con* RNAi with DA1-PN colabeling	*UAS-Dcr2, UAS-mCD8-GFP / w or y*	*R24A10-QF2, QUAS-mtdTomato-HA / UAS-con-RNAi (VDRC17898)*	*R78H05-p65.AD, R22E04-GAL4.DBD / +*
	Kek1 overexpression with VA1v-PN colabeling	*UAS-Dcr2 / w or y*	*VT003280-LexA, lexAop-rCD2::RFP-p10.UAS-mCD8::GFP-p10 / UAS-Kek1*	*R78H05-p65.AD, R22E04-GAL4.DBD / +*
	Fili overexpression with VA1v-PN colabeling	*UAS-Dcr2 / w or y*	*VT003280-LexA, lexAop-rCD2::RFP-p10.UAS-mCD8::GFP-p10 / +*	*R78H05-p65.AD, R22E04-GAL4.DBD / UAS-Fili*
	Kirre overexpression with VA1v-PN colabeling	*UAS-Dcr2 / UAS-Kirre*	*VT003280-LexA, lexAop-rCD2::RFP-p10.UAS-mCD8::GFP-p10 / +*	*R78H05-p65.AD, R22E04-GAL4.DBD / +*
	*Ptp10D* RNAi with VA1v-PN colabeling	*UAS-Dcr2 / w or y*	*VT003280-LexA, lexAop-rCD2::RFP-p10.UAS-mCD8::GFP-p10 / +*	*R78H05-p65.AD, R22E04-GAL4.DBD / UAS-Ptp10D-RNAi*
	*ten-a* RNAi with VA1v-PN colabeling	*UAS-Dcr2 / w or y*	*VT003280-LexA, lexAop-rCD2::RFP-p10.UAS-mCD8::GFP-p10 / +*	*R78H05-p65.AD, R22E04-GAL4.DBD / UAS-Ten-a-RNAi*
	Ten-m overexpression with VA1v-PN colabeling	*UAS-Dcr2 / w or y*	*VT003280-LexA, lexAop-rCD2::RFP-p10.UAS-mCD8::GFP-p10 / +*	*R78H05-p65.AD, R22E04-GAL4.DBD / UAS-Ten-m*
	*klg* RNAi with VA1v-PN colabeling	*UAS-Dcr2 / w or y*	*VT003280-LexA, lexAop-rCD2::RFP-p10.UAS-mCD8::GFP-p10 / +*	*R78H05-p65.AD, R22E04-GAL4.DBD / UAS-klg-RNAi*
	*con* RNAi with VA1v-PN colabeling	*UAS-Dcr2 / w or y*	*VT003280-LexA, lexAop-rCD2::RFP-p10.UAS-mCD8::GFP-p10 / UAS-con-RNAi*	*R78H05-p65.AD, R22E04-GAL4.DBD / +*
Figure 2				
b	*R+* x2 with DA1-PN colabeling	*UAS-Dcr2, UAS-mCD8-GFP / w or y*	*R24A10-QF2, QUAS-mtdTomato-HA / UAS-Kek1*	*R78H05-p65.AD, R22E04-GAL4.DBD / UAS-Fili*
	*R+* x3 with DA1-PN colabeling	*UAS-Dcr2, UAS-mCD8-GFP / UAS-Kirre*	*R24A10-QF2, QUAS-mtdTomato-HA / UAS-Kek1*	*R78H05-p65.AD, R22E04-GAL4.DBD / UAS-Fili*
	Δ*A* x2 with DA1-PN colabeling	*UAS-Dcr2, UAS-mCD8-GFP / w or y*	*R24A10-QF2, QUAS-mtdTomato-HA / UAS-con-RNAi*	*R78H05-p65.AD, R22E04-GAL4.DBD / UAS-ten-a-RNAi*
	Δ*A* x2 & *R-* with DA1-PN colabeling	*UAS-Dcr2, UAS-mCD8-GFP / w or y*	*R24A10-QF2, QUAS-mtdTomato-HA / UAS-con-RNAi*	*R78H05-p65.AD, R22E04-GAL4.DBD / UAS-ten-a-RNAi, UAS-Ptp10D-RNAi*
	Δ*A* x2 & *R+* x2 with DA1-PN colabeling	*UAS-Dcr2, UAS-mCD8-GFP / w or y*	*R24A10-QF2, QUAS-mtdTomato-HA / UAS-con-RNAi, UAS-Kek1*	*R78H05-p65.AD, R22E04-GAL4.DBD / UAS-ten-a-RNAi, UAS-Fili*
	*R+* x2 & *R-* with DA1-PN colabeling	*UAS-Dcr2, UAS-mCD8-GFP / w or y*	*R24A10-QF2, QUAS-mtdTomato-HA / UAS-Kek1*	*R78H05-p65.AD, R22E04-GAL4.DBD / UAS-Fili, UAS-Ptp10D-RNAi*
	*R+* x2 with VA1v-PN colabeling	*UAS-Dcr2, UAS-mCD8-GFP / w or y*	*VT003280-LexA, lexAop-rCD2::RFP-p10.UAS-mCD8::GFP-p10 / UAS-Kek1*	*R78H05-p65.AD, R22E04-GAL4.DBD / UAS-Fili*
	*R+* x3 with VA1v-PN colabeling	*UAS-Dcr2, UAS-mCD8-GFP / UAS-Kirre*	*VT003280-LexA, lexAop-rCD2::RFP-p10.UAS-mCD8::GFP-p10 / UAS-Kek1*	*R78H05-p65.AD, R22E04-GAL4.DBD / UAS-Fili*
	Δ*A* x2 with VA1v-PN colabeling	*UAS-Dcr2, UAS-mCD8-GFP / w or y*	*VT003280-LexA, lexAop-rCD2::RFP-p10.UAS-mCD8::GFP-p10 / UAS-con-RNAi*	*R78H05-p65.AD, R22E04-GAL4.DBD / UAS-ten-a-RNAi*
	Δ*A* x2 *& R-* with VA1v-PN colabeling	*UAS-Dcr2, UAS-mCD8-GFP / w or y*	*VT003280-LexA, lexAop-rCD2::RFP-p10.UAS-mCD8::GFP-p10 / UAS-con-RNAi*	*R78H05-p65.AD, R22E04-GAL4.DBD / UAS-ten-a-RNAi, UAS-Ptp10D-RNAi*
	Δ*A* x2 *& R+* x2 with VA1v-PN colabeling	*UAS-Dcr2, UAS-mCD8-GFP / w or y*	*VT003280-LexA, lexAop-rCD2::RFP-p10.UAS-mCD8::GFP-p10 / UAS-con-RNAi, UAS-Kek1*	*R78H05-p65.AD, R22E04-GAL4.DBD / UAS-ten-a-RNAi, UAS-Fili*
	*R+* x2 & *R-* with VA1v-PN colabeling	*UAS-Dcr2, UAS-mCD8-GFP / w or y*	*VT003280-LexA, lexAop-rCD2::RFP-p10.UAS-mCD8::GFP-p10 / UAS-Kek1*	*R78H05-p65.AD, R22E04-GAL4.DBD / UAS-Fili, UAS-Ptp10D-RNAi*
c	*R+* x2 & *R- & AA* x2 with DA1-PN colabeling	*UAS-Dcr2, UAS-mCD8-GFP / w or y*	*R24A10-QF2, QUAS-mtdTomato-HA / UAS-con-RNAi, UAS-Kek1*	*R78H05-p65.AD, R22E04-GAL4.DBD / UAS-ten-a-RNAi, UAS-Fili, UAS-Ptp10D-RNAi*
	*R+* x2 & *R- & AA* x2 with VA1v-PN and VAlv-ORN colabeling	*UAS-Dcr2, UAS-mCD8-GFP / w or y*	*VT003280-LexA, lexAop-rCD2::RFP-p10.UAS-mCD8::GFP-p10 / UAS-con-RNAi, UAS-Kek1, OR4 7b-ratCD2 (BDSC 9916)*	*R78H05-p65.AD, R22E04-GAL4.DBD / UAS-ten-a-RNAi, UAS-Fili, UAS-Ptp10D-RNAi*
Figure 3				
	Control	*UAS-Dcr2 / w*	*VT003280-LexA, UAS-IVS-myr-tdtomato (BDSC 32222) / VT003280-LexA*	*R78H05-p65.AD, R22E04-GAL4.DBD, LexAop-IVS-jGCaMP7b (BDSC 80915) / +*
	DA1-ORN rewired	*UAS-Dcr2 / w*	*VT003280-LexA, UAS-IVS-myr-tdtomato / UAS-con-RNAi, UAS-Kek1, VT003280-LexA*	*R78H05-p65.AD, R22E04-GAL4.DBD, LexAop-IVS-jGCaMP7b / UAS-ten-a-RNAi, UAS-Fili, UAS-Ptp10D-RNAi*
Figure 4				
	Control male	*UAS-Dcr2 /y*	*VT003280-LexA, UAS-IVS-myr-tdtomato (BDSC 32222) / VT003280-LexA*	*R78H05-p65.AD, R22E04-GAL4.DBD, LexAop-IVS-jGCaMP7b (BDSC 80915) / +*
	DA1-ORN rewired male	*UAS-Dcr2 /y*	*VT003280-LexA, UAS-IVS-myr-tdtomato / UAS-con-RNAi, UAS-Kek1, VT003280-LexA*	*R78H05-p65.AD, R22E04-GAL4.DBD, LexAop-IVS-jGCaMP7b / UAS-ten-a-RNAi, UAS-Fili, UAS-Ptp10D-RNAi*
Figure 5				
b, c	VA1d-PN & wild type	*UAS-Dcr2, UAS-mCD8-GFP / w or y*	*R20D10-QF2 (ref Lyu et al 2025), QUAS-mtdTomato-HA / +*	*R78H05-p65.AD, R31F09-GAL4.DBD (BDSC 68759) / +*
	VA1d-PN & CSP set 1	*UAS-Dcr2, UAS-mCD8-GFP / w or y*	*R20D10-QF2, QUAS-mtdTomato-HA / UAS-con-RNAi, UAS-Kek1*	*R78H05-p65.AD, R31F09-GAL4.DBD / UAS-Ptp10D-RNAi, UAS-Sema-2b-RNAi (BDSC 28932)*
	VA1d-PN & CSP set 2	*UAS-Dcr2, UAS-mCD8-GFP / UAS-Kirre*	*R20D10-QF2, QUAS-mtdTomato-HA / UAS-Kekl*	*R78H05-p65.AD, R31F09-GAL4.DBD / UAS-Ptp10D-RNAi, UAS-Ten-m-RNAi (BDSC 29390), UAS-Klg* ^17^
	VA1d-PN & CSP set 3	*UAS-Dcr2, UAS-mCD8-GFP / UAS-Ten-a (BDSC 41563)*	*R20D10-QF2, QUAS-mtdTomato-HA / +*	*R78H05-p65.AD, R31F09-GAL4.DBD / UAS-Sema-2b-RNAi*
	VA1v-PN & wild type	*UAS-Dcr2 / w or y*	*VT003280-LexA, lexAop-rCD2::RFP-p10.UAS-mCD8::GFP-p10 / +*	*R78H05-p65.AD, R31F09-GAL4.DBD / +*
	VA1v-PN & CSP set 1	*UAS-Dcr2 / w or y*	*VT003280-LexA, lexAop-rCD2::RFP-p10.UAS-mCD8::GFP-p10 / UAS-con-RNAi, UAS-Kek1*	*R78H05-p65.AD, R31F09-GAL4.DBD / UAS-Ptp10D-RNAi, UAS-Sema-2b-RNAi*
	VA1v-PN & CSP set 2	*UAS-Dcr2 / UAS-Kirre*	*VT003280-LexA, lexAop-rCD2::RFP-p10.UAS-mCD8::GFP-p10 / UAS-Kek1*	*R78H05-p65.AD, R31F09-GAL4.DBD / UAS-Ptp10D-RNAi, UAS-Ten-m-RNAi, UAS-Klg*
	VA1v-PN & CSP set 3	*UAS-Dcr2 / UAS-Ten-a*	*VT003280-LexA, lexAop-rCD2::RFP-p10.UAS-mCD8::GFP-p10 / +*	*R78H05-p65.AD, R31F09-GAL4.DBD / UAS-Sema-2b-RNAi*
	DC3-PN & wild type	*UAS-Dcr2, UAS-mCD8-GFP / w or y*	*R82E01-QF2 (ref Lyu et al 2025), QUAS-mtdTomato-HA / +*	*R78H05-p65.AD, R31F09-GAL4.DBD / +*
	DC3-PN & CSP set 1	*UAS-Dcr2, UAS-mCD8-GFP / w or y*	*R82E01-QF2, QUAS-mtdTomato-HA / UAS-con-RNAi, UAS-Kek1*	*R78H05-p65.AD, R31F09-GAL4.DBD / UAS-Pip10D-RNAi, UAS-Sema-2b-RNAi*
	DC3-PN & CSP set 2	*UAS-Dcr2, UAS-mCD8-GFP / UAS-Kirre*	*R82E01-QF2, QUAS-mtdTomato-HA / UAS-Kekl*	*R78H05-p65.AD, R31F09-GAL4.DBD / UAS-Ptp10D-RNAi, UAS-Ten-m-RNAi, UAS-Klg*
	DC3-PN & CSP set 3	*UAS-Dcr2, UAS-mCD8-GFP / UAS-Ten-a*	*R82E01-QF2, QUAS-mtdTomato-HA / +*	*R78H05-p65.AD, R31F09-GAL4.DBD / UAS-Sema-2b-RNAi*
	DL3-PN & wild type	*UAS-Dcr2 / w or y*	*R37H08-LexA (BDSC 52766), lexAop-rCD2::RFP-p10.UAS-mCD8::GFP-p10 / +*	*R78H05-p65.AD, R31F09-GAL4.DBD / +*
	DL3-PN & CSP set 1	*UAS-Dcr2 / w or y*	*R37H08-LexA, lexAop-rCD2::RFP-p10.UAS-mCD8::GFP-p10 / UAS-con-RNAi, UAS-Kek1*	*R78H05-p65.AD, R31F09-GAL4.DBD / UAS-Ptp10D-RNAi, UAS-Sema-2b-RNAi*
	DL3-PN & CSP set 2	*UAS-Dcr2 / UAS-Kirre*	*R37H08-LexA, lexAop-rCD2::RFP-p10.UAS-mCD8::GFP-p10 / UAS-Kek1*	*R78H05-p65.AD, R31F09-GAL4.DBD / UAS-Ptp10D-RNAi, UAS-Ten-m-RNAi, UAS-Klg*
	DL3-PN & CSP set 3	*UAS-Dcr2 / UAS-Ten-a*	*R37H08-LexA, lexAop-rCD2::RFP-p10.UAS-mCD8::GFP-p10 / +*	*R78H05-p65.AD, R31F09-GAL4.DBD / UAS-Sema-2b-RNAi*
d, e, j	controls	*UAS-Dcr2 / w*	*VT003280-LexA, UAS-IVS-myr-tdtomato / VT003280-LexA*	*R78H05-p65.AD, R31F09-GAL4.DBD, LexAop-IVS-jGCaMP7b / +*
	VA1d-ORN to VA1v-PN rewired	*UAS-Dcr2 / w*	*VT003280-LexA, UAS-IVS-myr-tdtomato / VT003280-LexA, UAS-con-RNAi, UAS-Kek1*	*R78H05-p65.AD, R31F09-GAL4.DBD, LexAop-IVS-jGCaMP7b / UAS-Ptp10D-RNAi, UAS-Sema-2b-RNAi*
f, g, k	controls	*UAS-Dcr2, QUAS-GCaMP8m (this work) / +*	*R82E01-QF2, UAS-IVS-myr-tdtomato / R82E01-QF2*	*R78H05-p65.AD, R31F09-GAL4.DBD, QUAS-GCaMP8m (this work) / +*
	VA1d-ORN to DC3-PN rewired	*UAS-Dcr2, QUAS-GCaMP8m / UAS-Kirre*	*R82E01-QF2, UAS-IVS-myr-tdtomato / UAS-Kekl, R82E01-QF2*	*R78H05-p65.AD, R31F09-GAL4.DBD, QUAS-GCaMP8m / UAS-Ptp10D-RNAi, UAS-Ten-m-RNAi, UAS-Klg*
h, i, l	controls	*UAS-Dcr2 / w*	*R3 7H08-LexA, UAS-IVS-myr-tdtomato / R37H08-LexA*	*R78H05-p65.AD, R31F09-GAL4.DBD, LexAop-IVS-jGCaMP7b / +*
	VA1d-ORN to DL3-PN rewired	*UAS-Dcr2 / UAS-Ten-a*	*R3 7H08-LexA, UAS-IVS-myr-tdtomato / R37H08-LexA*	*R78H05-p65.AD, R31F09-GAL4.DBD, LexAop-IVS-jGCaMP7b / UAS-Sema-2b-RNAi*
Extended Data Figure. 1				
	DA1-ORN	*UAS-mCD8-GFP / w or y*	+	*R78H05-p65.AD, R22E04-GAL4.DBD / +*
Extended Data Figure. 2				
c		*UAS-Dcr2 / w or y*	*Mz19-GAL4 (BDSC 34497), QUAS_mCD8_GFP (BDSC 30002), Or47b_rCD2, Or88a_mtdTomato (BDSC 64070)/ +*	+
d		*UAS-Dcr2 / w or y*	*Mz19-GAL4, QUAS_mCD8_GFP, Or47b_rCD2, Or88a_mtdTomato / UAS-con-RNAi*	+
e		*UAS-Dcr2, UAS-mCD8-GFP / w or y*	*R20D10-QF2, QUAS-mtdTomato-HA / +*	*R78H05-p65.AD, R31F09-GAL4.DBD / +*
f		*UAS-Dcr2, UAS-mCD8-GFP / w or y*	*R20D10-QF2, QUAS-mtdTomato-HA / +*	*R78H05-p65.AD, R31F09-GAL4.DBD / UAS-Klg*
Extended Data Figure. 5				
	No *ptp10D* RNAi with DA1-PN colabeling	*UAS-Dcr2, UAS-mCD8-GFP / w or y*	*R24A10-QF2, QUAS-mtdTomato-HA / UAS-con-RNAi, UAS-Kek1*	*R78H05-p65.AD, R22E04-GAL4.DBD / UAS-ten-a-RNAi, UAS-Fili*
	No *ten-a* RNAi with DA1-PN colabeling	*UAS-Dcr2, UAS-mCD8-GFP / w or y*	*R24A10-QF2, QUAS-mtdTomato-HA / UAS-con-RNAi, UAS-Kek1*	*R78H05-p65.AD, R22E04-GAL4.DBD / UAS-Fili, UAS-Ptp10D-RNAi*
	No *con* RNAi with DA 1 - PN colabeling	*UAS-Dcr2, UAS-mCD8-GFP / w or y*	*R24A10-QF2, QUAS-mtdTomato-HA / UAS-Kek1*	*R78H05-p65.AD, R22E04-GAL4.DBD / UAS-ten-a-RNAi, UAS-Fili, UAS-Ptp10D-RNAi*
	No Kek1 OE with DA1-PN colabeling	*UAS-Dcr2, UAS-mCD8-GFP / w or y*	*R24A10-QF2, QUAS-mtdTomato-HA / UAS-con-RNAi*	*R78H05-p65.AD, R22E04-GAL4.DBD / UAS-ten-a-RNAi, UAS-Fili, UAS-Ptp10D-RNAi*
	No Fili OE with DA1-PN colabeling	*UAS-Dcr2, UAS-mCD8-GFP / w or y*	*R24A10-QF2, QUAS-mtdTomato-HA / UAS-con-RNAi, UAS-Kek1*	*R78H05-p65.AD, R22E04-GAL4.DBD / UAS-ten-a-RNAi, UAS-Ptp10D-RNAi*
	No *ptp10D* RNAi with VA1v-PN colabeling	*UAS-Dcr2, UAS-mCD8-GFP / w or y*	*VT003280-LexA, lexAop-rCD2::RFP-p10.UAS-mCD8::GFP-p10 / UAS-con-RNAi, UAS-Kek1*	*R78H05-p65.AD, R22E04-GAL4.DBD / UAS-ten-a-RNAi, UAS-Fili*
	No *ten-a* RNAi with VA1v-PN colabeling	*UAS-Dcr2, UAS-mCD8-GFP / w or y*	*VT003280-LexA, lexAop-rCD2::RFP-p10.UAS-mCD8::GFP-p10 / UAS-con-RNAi, UAS-Kek1*	*R78H05-p65.AD, R22E04-GAL4.DBD / UAS-Fili, UAS-Ptp10D-RNAi*
	No *con* RNAi with VA1v-PN colabeling	*UAS-Dcr2, UAS-mCD8-GFP / w or y*	*VT003280-LexA, lexAop-rCD2::RFP-p10.UAS-mCD8::GFP-p10 / UAS-Kek1*	*R78H05-p65.AD, R22E04-GAL4.DBD / UAS-ten-a-RNAi, UAS-Fili, UAS-Ptp10D-RNAi*
	No Kek1 OE with VA1v-PN colabeling	*UAS-Dcr2, UAS-mCD8-GFP / w or y*	*VT003280-LexA, lexAop-rCD2::RFP-p10.UAS-mCD8::GFP-p10 / UAS-con-RNAi*	*R78H05-p65.AD, R22E04-GAL4.DBD / UAS-ten-a-RNAi, UAS-Fili, UAS-Ptp10D-RNAi*
	No Fili OE with VA1v-PN colabeling	*UAS-Dcr2, UAS-mCD8-GFP / w or y*	*VT003280-LexA, lexAop-rCD2::RFP-p10.UAS-mCD8::GFP-p10 / UAS-con-RNAi, UAS-Kek1*	*R78H05-p65.AD, R22E04-GAL4.DBD / UAS-ten-a-RNAi, UAS-Ptp10D-RNAi*
	5-CSP manipulation	*Same as* Fig. 2c		
Extended Data Figure. 6				
	rewired male with DA1-PN colabeling	*UAS-Dcr2, UAS-mCD8-GFP / y*	*R24A10-QF2, QUAS-mtdTomato-HA / UAS-con-RNAi, UAS-Kek1*	*R78H05-p65.AD, R22E04-GAL4.DBD / UAS-ten-a-RNAi, UAS-Fili, UAS-Ptp10D-RNAi*
	rewired male with VA1v-PN colabeling	*UAS-Dcr2, UAS-mCD8-GFP / y*	*VT003280-LexA, lexAop-rCD2::RFP-p10.UAS-mCD8::GFP-p10 / UAS-con-RNAi, UAS-Kek1*	*R78H05-p65.AD, R22E04-GAL4.DBD / UAS-ten-a-RNAi, UAS-Fili, UAS-Ptp10D-RNAi*
	rewired female with DA1-PN colabeling	*UAS-Dcr2, UAS-mCD8-GFP / w*	*R24A10-QF2, QUAS-mtdTomato-HA / UAS-con-RNAi, UAS-Kek1*	*R78H05-p65.AD, R22E04-GAL4.DBD / UAS-ten-a-RNAi, UAS-Fili, UAS-Ptp10D-RNAi*
	rewired female with VA1v-PN colabeling	*UAS-Dcr2, UAS-mCD8-GFP / w*	*VT003280-LexA, lexAop-rCD2::RFP-p10.UAS-mCD8::GFP-p10 / UAS-con-RNAi, UAS-Kek1*	*R78H05-p65.AD, R22E04-GAL4.DBD / UAS-ten-a-RNAi, UAS-Fili, UAS-Ptp10D-RNAi*
Extended Data Figure. 7				
	Control male	*UAS-Dcr2 /y*	*VT003280-LexA, UAS-IVS-myr-tdtomato (BDSC 32222) / VT003280-LexA*	*R78H05-p65.AD, R22E04-GAL4.DBD, LexAop-IVS-jGCaMP7b (BDSC 80915) / +*
	DA1-ORN rewired male	*UAS-Dcr2 /y*	*VT003280-LexA, UAS-IVS-myr-tdtomato / UAS-con-RNAi, UAS-Kek1, VT003280-LexA*	*R78H05-p65.AD, R22E04-GAL4.DBD, LexAop-IVS-jGCaMP7b / UAS-ten-a-RNAi, UAS-Fili, UAS-Ptp10D-RNAi*
	female	*Canton-S (BDSC 64349)*		
Extended Data Figure. 9				
a	VA1v-PN & CSP set 1 without trajectory manipulation	*UAS-Dcr2 / w or y*	*VT003280-LexA, lexAop-rCD2::RFP-p10.UAS-mCD8::GFP-p10 / UAS-con-RNAi, UAS-Kek1*	*R78H05-p65.AD, R31F09-GAL4.DBD / UAS-Ptp10D-RNAi*
b	DL3-PN & CSP set 3 without trajectory manipulation	*UAS-Dcr2 / UAS-Ten-a*	*R37H08-LexA, lexAop-rCD2::RFP-p10.UAS-mCD8::GFP-p10 / +*	*R78H05-p65.AD, R31F09-GAL4.DBD / +*
c, d	Same as Fig. 5b, but without *Sema-2b* RNAi in groups that do not have trajectory manipulation
